# Screening of a Combinatorial Library of Triazine-Scaffolded Dipeptide-Mimic Affinity Ligands to Bind Plasmid DNA

**DOI:** 10.3390/molecules30163423

**Published:** 2025-08-19

**Authors:** João F. R. Belchior, Gabriel A. Monteiro, D. Miguel Prazeres, M. Ângela Taipa

**Affiliations:** 1 iBB—Institute for Bioengineering and Biosciences, and Associate Laboratory i4HB—Institute for Health and Bioeconomy at Instituto Superior Técnico, Universidade de Lisboa, Av. Rovisco Pais, 1049-001 Lisboa, Portugalmiguelprazeres@tecnico.ulisboa.pt (D.M.P.); 2 Bioengineering Department, Instituto Superior Técnico, Universidade de Lisboa, Av. Rovisco Pais, 1049-001 Lisboa, Portugal

**Keywords:** triazine-scaffolded biomimetic ligands, synthetic combinatorial ligand library, high-throughput screening, affinity chromatography, plasmid purification

## Abstract

Plasmid DNA (pDNA) purification plays a key role in the development of vaccines and gene therapies. Affinity chromatography stands out as a promising method for plasmid purification, leveraging a range of biological and synthetic ligands to achieve selectivity. This study investigates the potential of a synthetic ligand library consisting of triazine-based bifunctional compounds designed to mimic the side chains of amino acids that are known to bind nucleic acids. A high-throughput screening method was employed to assess the binding ability of 158 ligands within the library to single-stranded, FITC-labeled homo-oligonucleotides (G and T), each comprising 20 nucleotides, under both hydrophilic and hydrophobic conditions. High-affinity ligands were identified for both T and G oligonucleotides. Follow-up microscale chromatographic screening uncovered some false positives from the initial FITC-based screening, narrowing the selection to 22 ligands for further investigation. In the next phase of the study, the binding affinity of these ligands towards double-stranded oligonucleotides (AT and CG) was assessed. Ligand 1/2, a mimic of Ala-Lys or Gly-Lys, and ligand 2/3, a mimic of Lys-Tyr, were chosen as initial candidates for evaluating plasmid DNA purification from an *Escherichia coli* crude extract. The results obtained with 0.4 M ammonium sulfate in 20 mM Tris-HCl (pH 8.0) as the binding buffer were similar to those observed when purifying plasmid DNA from *E. coli* clarified lysates by hydrophobic interaction chromatography. The affinity resins retained RNA, while the less hydrophobic plasmid DNA was excluded in the initial fractions. Future research will be directed towards exploring the potential of the most promising ligands to separate pDNA isoforms.

## 1. Introduction

Nucleic acid therapeutics hold the promise to transform the biopharmaceutical industry, offering highly effective vaccines and innovative treatments for cancer and genetic disorders. The significant progress made in the past decade in developing both DNA- and RNA-based biotherapeutics is exemplified by the successful creation of mRNA vaccines for protection against COVID-19 [1]. Plasmids, in particular, are widely used across the current gene and cell therapy industry. As biologicals, plasmids deliver genetic material to target cells in patients (e.g., as DNA vaccines) or act as carriers for the molecular components of gene editing systems. Additionally, plasmids function as crucial raw materials in the production of engineered cell therapies (such as CAR-T cells) and other biologics, including viral vectors and mRNA [2,3,4].

Plasmids used in this context contain both the eukaryotic elements needed for the expression of the gene of interest in the patient’s cells and the prokaryotic segments required for its replication in the producing bacterial host. One of the intrinsic features of plasmid DNA (pDNA) is its ability to exist in multiple topological isoforms. Among these, the supercoiled isoform (sc), which is characterized by a fully intact, tightly twisted DNA double helix without free ends, is preferred in gene therapy applications due to its superior efficiency in transcription by host cells. Although the FDA recommends that plasmid preparations should contain >80% of the sc isoform, industry guidelines usually target >90–95% [4,5]. During pDNA extraction and purification, exposure to significant shear stress can damage the phosphodiester backbone, causing single-strand nicks that relax the supercoiled structure and generate open circular (oc) isoforms. Similar degradation may also occur if the purified plasmid is left at room temperature within a pH range of 7.5 to 8.5 [6].

Large-scale production of pDNA relies on its replication in *Escherichia coli* cells. The subsequent downstream processing of pDNA from *E. coli* biomass is an engineering challenge, which has been largely resolved by linking a sequence of operations such as alkaline lysis, tangential flow filtration, and chromatography. These steps, in various combinations, yield pDNA with residual host impurities (e.g., genomic DNA, RNA, host cell proteins, and endotoxins) at levels compliant with regulatory standards that minimize adverse reactions in the human host [5].

Various chromatographic techniques, individually or in combination, have been incorporated into processes for producing therapeutic pDNA. However, modalities such as size exclusion (SEC), anion exchange (AEC), hydrophobic interaction (HIC), and multimodal (MMC) chromatography often lack selectivity, particularly in terms of pDNA isoform separation. Affinity chromatography, on the other hand, has a clear potential to deliver higher specificity [7]. Examples can be found in the literature that explore affinity interactions between pDNA or impurities with ligands such as triple-helix-forming oligonucleotides [8], boronated binding domains [9], berenil [10], Cibacron Blue F3GA [11], and amino acids (e.g., histidine, arginine, lysine) [12,13,14,15,16]. The latter case explores the binding of amino acids to nucleic acids through a combination of electrostatic attraction between positively charged side chains and negatively charged phosphate groups, as well as van der Waals forces and hydrogen bonding with functional groups in nucleotides [17]. In this context, designing synthetic molecules that replicate key properties of specific amino acids, peptides, or proteins—while also introducing additional functional features—offers the potential to create ligands with enhanced selectivity. Triazine-scaffolded synthetic affinity ligands are chemically defined, inexpensive [18,19], and more easily produced than amino acid ligands [20]. In addition, such synthetic ligands resist degradation caused by chemical and biological agents, being scalable, durable, and reusable over multiple cycles. They are also generally not toxic *per se,* nor are the respective column leachates. Their exceptional stability allows harsh elution conditions as well as in situ cleaning and sterilization procedures. Triazine-scaffolded synthetic affinity ligands can mimic the function of whole proteins and are designated as ‘biomimetic ligands’ [21].

**Figure 1:**
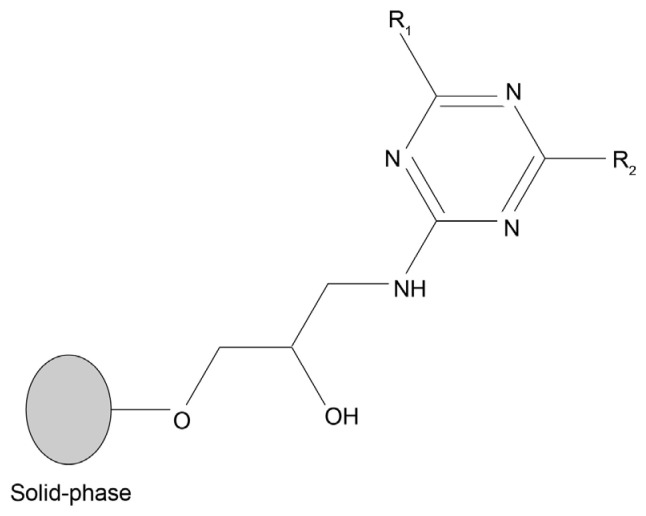
General structure of triazine-based dipeptide-mimic affinity ligands. Each ligand is bound to agarose (solid-phase) via a spacer arm. Substituents in the R1 and R2 positions consist of various aliphatic and aromatic amines that mimic amino acid side chains. Adapted from [21].

A solid-phase combinatorial library of triazine-scaffolded ligands, mimicking 11 of the 20 essential amino acids, was synthesized as described in [22]. The general structure of each ligand is represented in Figure 1. By varying the structural composition of the substituents in the R1 and R2 positions on the triazine ring, different amino acid combinations can be mimicked.

A high-throughput qualitative screening was performed using fluorescein isothiocyanate (FITC)-labeled single-stranded oligonucleotides (Poly G and Poly T) to randomly screen the ligand library for binding DNA molecules, under both hydrophilic and hydrophobic conditions. Binding ligands were re-evaluated by micro-scale affinity chromatography, using single-stranded homo-oligonucleotides (Poly G and Poly T). After this screening step, 22 ligands were selected and assessed for their ability to recognize and bind double-stranded DNA sequences (hybridized homo-oligonucleotides (AT and CG)). Two lead compounds were further tested in the purification of plasmid pVAX1/LacZ, which was used as a model for the purification of real samples. The entire study showcases a robust and efficient approach for screening a large combinatorial ligand library to bind DNA molecules and to identify potential candidates for plasmid DNA purification from *Escherichia coli* crude lysates.

## 2. Results

### 2.1. Preliminary Binding Assessment

A combinatorial library of protein-mimic affinity ligands, synthesized as described in Roque et al. [22], was randomly screened to bind nucleic acid molecules. This synthetic library is based on a triazine scaffold with amino substituents at two defined positions (Figure 1). Variation in the structural composition of these amino substituents enables the mimicry of diverse amino acid side chains. The synthesized ligands can act as ‘artificial dipeptides’, and hence interact with DNA sequences, since nucleic acids are known to have affinity towards amino acid-containing structures, such as leucine zippers, and other protein motifs [23].

**Figure 2:**
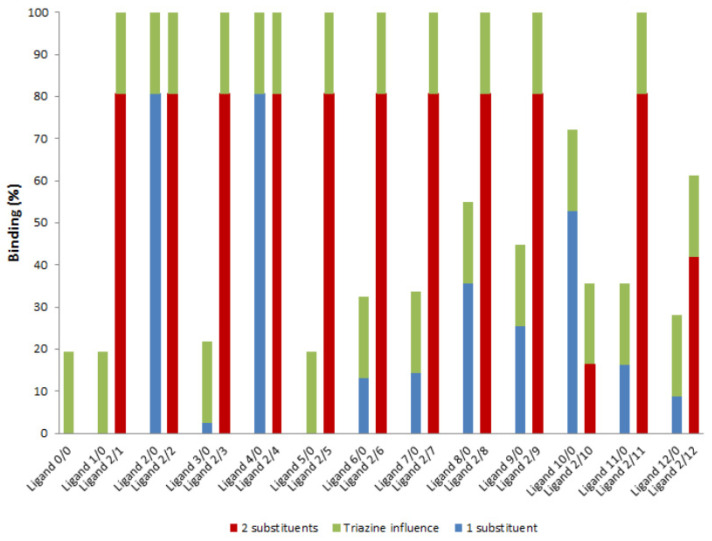
Binding profile of different ligand-derivatized resins R1/0 (blue bars) and 2/R2 (red bars). The DNA molecules used were a mixture of single-stranded DNA oligonucleotides (available in the laboratory) containing 27 nucleotides (MRS2rev and MRS2fwd). Microscale chromatographic assays were performed as described in Materials and Methods (Section 3.7). The green bars represent the percentage of binding potentially originating from the triazine scaffold.

According to the literature, lysine, arginine, and histidine are the amino acids with the highest affinity towards nucleic acids [17]. The ligand library used in the screening process (Table 1) included ligands with amino substituents that mimic one of these amino acids, lysine—1,5-diaminopentane (substituent 2) and m-xylylenediamine (substituent 4). The first set of experiments was performed with ligands containing substituent 2 combined with all the other amino substituents (ligands 2/R2). The ability of these ligands to bind DNA molecules was assessed by affinity chromatographic assays with ligand’s derivatized agarose (solid-phase ligands). The DNA molecules used were single-stranded DNA oligonucleotides (available in the laboratory) containing 27 nucleotides (MRS2re and MRS2fwd, described in the Materials and Methods). Similar assays were performed with triazine ligands containing only one of the substituents in the R1 position (1/0, 2/0, 3/0, 4/0, 5/0, 6/0, 7/0, 8/0, 9/0, 10/0, 11/0 and 12/0) and with the aminated triazine scaffold, in which R1 and R2 positions were substituted by NH_3_ groups (ligand 0/0) as a control (Figure 2). All experiments were performed under hydrophilic conditions, using 20 mM Tris-HCl pH 8.0 as the binding buffer. The results are shown in Figure 2.

The results shown in Figure 2 indicate that the triazine ring can contribute up to 20% to the binding yield. The triazine molecule can interact with DNA through nitrogen structural atoms. In this preliminary assessment, it was presumed that the contribution of the triazine ring to the overall binding yield would be similar for all the tested ligands. However, depending on the amino compounds, the interaction with the triazine scaffold may be less pronounced when certain amino substituents are present at the R1 and R2 positions.

Various types of interactions can occur between DNA and amino acid residues (electrostatic interactions, van der Waals interactions, hydrogen bonds, and water-mediated bonds) [17]. Some amino acids bind not only to one nucleic acid base, but also to a specific sequence, forming complexes. DNA-protein interactions are influenced by both the nucleotide bases and the sugar–phosphate backbone, the former of which can contribute to binding specificity and affinity [17,23].

When analyzing the results with only one substituent, ligands 2/0 and 4/0 stand out as those with higher binding capacities. This outcome was anticipated, as both ligands mimic the amino acid lysine, which is known from the literature to have a high affinity for DNA. Lysine is particularly effective at forming hydrogen bonds, as well as van der Waals and water-mediated interactions. Hydrogen bonds confer specificity to amino acids–DNA binding, particularly in the case of complex interactions, where more than one interaction occurs with the DNA sequence [17]. As lysine is a multiple-hydrogen-donor amino acid, it can perform complex interactions, particularly with guanine, as demonstrated in previous studies [23]. In addition, ligands 2/0 and 4/0 are, like lysine, positively charged and can also promote electrostatic interactions with the negatively charged phosphate groups of the DNA molecule.

Ligand 10/0, mimicking aspartic acid, also exhibited a high capacity for binding DNA oligonucleotides (binding yield ~ 52%). Aspartic acid is considered a double hydrogen bond acceptor, making it well-suited to forming hydrogen bonds with DNA [17]. Water-mediated bonds can also contribute to this interaction since the pair Asp-A is one of the most favored amino acid-base pairs in water-mediated interactions. In the case of aspartic acid, the ability to bind DNA by water-mediated interactions is mostly due to its short side chain [23]. However, the fact that the ligand is negatively charged at pH 8.0 prevents 100% binding to DNA molecules due to unfavorable electrostatic interactions.

Ligand 8/0, a mimic of asparagine and glutamine, exhibited a binding yield of ~35%. Glutamine and asparagine are acceptors and donors in hydrogen bonds and are frequently found in amino acid-base pairs, especially with the nucleotide base adenine [24]. Gln and Asn can form two hydrogen bonds with adenine, involving the carbonyl oxygen of the Gln/Asn side chain (acceptor) and nitrogen N6 of adenine (donor). These two amino acids are also commonly involved in the formation of stair motifs, making cation-π interactions [23]. Ligand 8/0 should have analogous properties. Additionally, its aromatic ring enables further stacking interactions with the DNA molecule.

Ligand 9/0 displayed a binding yield of ~25%. This ligand mimics threonine, which, like asparagine and glutamine, is both a hydrogen bond acceptor and donor. However, Thr is not able to form complex hydrogen bonds due to the steric hindrance of its methyl group [17]. Despite this, threonine is one of the amino acids most involved in van der Waals interactions. The pairing of threonine with adenine constitutes one of the most favored in water-mediated interactions [25].

Ligands 3/0, 6/0, 7/0, 11/0, and 12/0, mimics of tyrosine, leucine, glutamic acid, isoleucine, and glutamine, respectively, exhibited low binding yields (less than 20%). These amino acids are usually disfavored in DNA–amino acid interactions, except glutamine. Although both ligands 8/0 and 12/0 are analogs of glutamine, ligand 8/0 is preferred due to its aromatic ring, which, as mentioned earlier, can have a stacking effect on the DNA bases, enhancing the interaction with DNA molecules. The other ligands (3/0, 6/0, 7/0, and 11/0) are less effective at forming hydrogen bonds or van der Waals interactions [26], which likely explains their weak binding to the DNA oligonucleotides. Ligands 1/0 and 5/0 also did not bind to the tested oligonucleotides. Ligand 1/0 mimics both alanine and glycine, while ligand 5/0 mimics phenylalanine. None of these three amino acids shows a strong preference for binding to nucleotides, nor do they demonstrate any affinity for a specific DNA base [27,28].

The assessment of triazine-based ligands for binding DNA, where one arm of the triazine scaffold mimics Lys (R1) and the second position (R2) features different substituents, revealed that in most cases, as expected, binding significantly improved (compared to mono-substituted ligands), often achieving nearly 100% DNA binding. An exception to this behavior was noted in the case of R2 substitution with compound 10 (yielding 2/10). Amine 10 mimics aspartic acid, thereby carrying a negative charge. In contrast, the lysine mimic (substituent 2) is positively charged. The combination of these two substituents is likely to result in a neutral charge effect, which may inhibit DNA binding under the conditions tested. Ligand 2/12 also exhibited a moderate binding yield (~63.8%), possibly due to challenging interactions with the DNA molecules caused by steric hindrance from the two amino substituents on the triazine ring.

### 2.2. Screening with FITC-Labeled Poly T and Poly G Oligonucleotides

**Figure 3:**
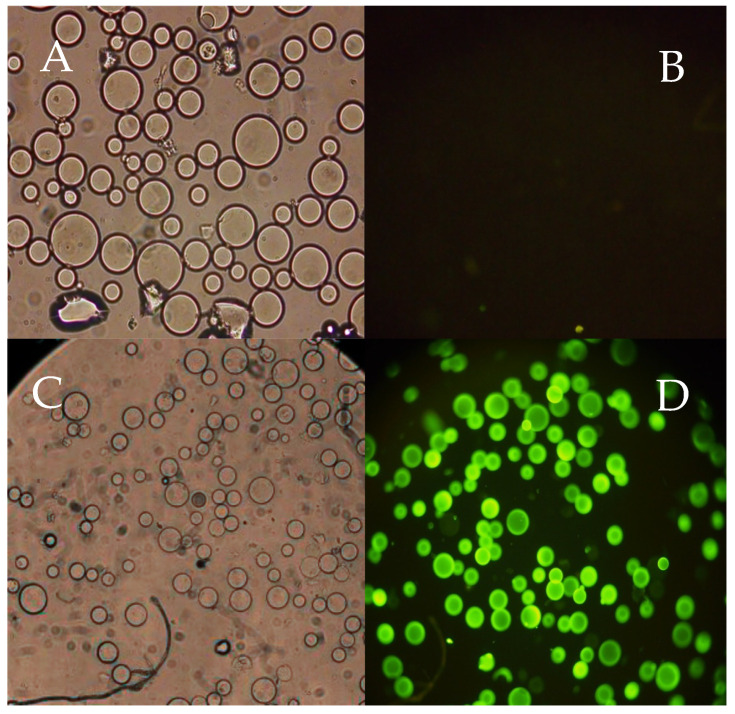
Images of affinity resins containing ligand 0/0 (negative control) and 2/2 (positive control) after incubation with a FITC-labeled single-stranded oligonucleotide (FITC-Poly T) in hydrophilic conditions (20 mM Tris HCl pH 8.0). (**A**) 0/0 under bright-field with a magnification of 20×; (**B**) 0/0 under fluorescent FITC emission with a magnification of 20×; (**C**) 2/2 (strong binder) under bright-field with a magnification of 10×; (**D**) under fluorescent FITC emission with a magnification of 10×.

Chromatography is the most reliable approach for screening ligand libraries, particularly for solid-phase synthesized libraries where the ligands are already immobilized. However, the scale of this process evaluation is usually limited, depending on the library size and the mass of target molecules available. Following a preliminary binding assessment of several ligands using a microscale chromatography assay, the ligand library was randomly screened for DNA binding using a fluorescence-based, high-throughput method similar to the one described in [29]. The resins were distributed in 96-well microplates, allowing for the simultaneous screening of multiple ligands (up to 96 per plate). After 15 min of incubation with target FITC-labeled molecules in the dark, the fluorescence emission in each well was examined under a fluorescence microscope and photographed, ensuring a robust qualitative evaluation of each ligand’s binding capacity. Using this screening methodology, ligands can be classified as strong binders, binders, or non-binders based on a qualitative criterion related to the fluorescence intensity of resin beads after incubation with the FCT-labeled targets under identical conditions. A preliminary test was run to establish the difference between a non-binder and a strong binder ligand. Ligand 0/0 was used as a non-binder (negative control) and ligand 2/2 as a strong binder ligand (positive control). After 15 min of incubation with a FITC-labeled single-stranded oligonucleotide (FITC-Poly T) in hydrophilic conditions (20 mM Tris HCl pH 8.0), both ligand-derivatized resins were visualized using a fluorescence microscope under bright-field and fluorescence illumination. The results obtained are depicted in Figure 3.

As shown in Figure 3, the negative control agarose beads observed in the well under bright-field (Figure 3A), are not visible when imaged under fluorescent conditions (Figure 3B). This confirms that the tested FITC-labeled oligonucleotide did not bind to the 0/0 ligand and was removed in the washing steps. Figure 3C,D, on the other hand, show the behavior of a strong binding ligand, as all the beads imaged under bright-field were also detected after FITC fluorescence excitation (at λ = 490 nm) and emission (at λ = 525 nm). This indicates that, despite the washing steps, the binding between the FITC-labeled oligonucleotide and the ligand on the derivatized beads remains strong.

**Figure 4:**
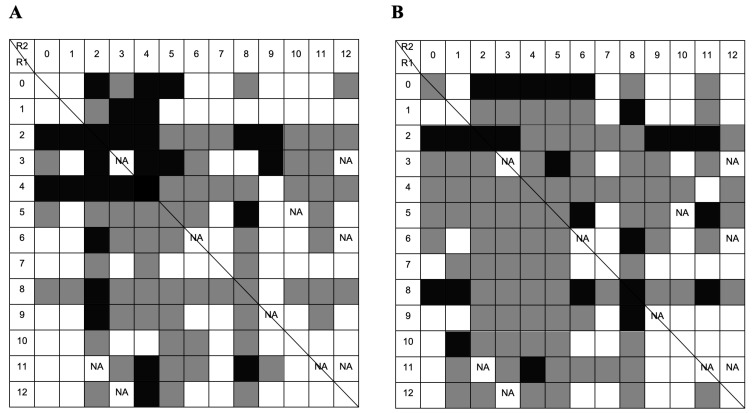
Summary of the FITC-based screening with 20 nt homo-oligonucleotide Poly T in: (**A**) hydrophilic conditions using 20 mM Tris-HCl pH 8.0 as the binding buffer; (**B**) hydrophobic conditions using 0.4 M ammonium sulfate in 20 mM Tris-HCl pH 8.0 as the binding buffer. Black squares indicate strong binders, gray squares indicate binders, and white squares indicate non-binders. NA: ligand not available. The line separates ligands that are obtained by adding the same substituents in reverse order on solid-phase synthesis [21].

FITC-labeled single-stranded homo-oligonucleotides (Poly T and Poly G), with 20 nucleotides (nt) each, were used as model DNA molecules for the screening of the combinatorial library under different binding conditions (hydrophilic and hydrophobic). Although the initial library consisted of 169 ligands, a few amines were not readily available and were excluded from the experiments. In total, 158 ligand-derivatized resins were screened. The following ligands were not tested: 3/3, 3/12, 5/10, 6/6, 6/12, 9/9, 11/2, 11/11, 11/12, and 12/3. The summary of the fluorescent-based screening with a FITC-labeled Poly T homo-oligonucleotide (FITC-Poly T) under hydrophilic and hydrophobic environments is schematically represented in Figure 4.

The results in Figure 4 show that 27 out of 158 ligands strongly bound to Poly T, whereas 68 out of 158 ligands did not exhibit any binding to the oligonucleotide. Most amino acid–nucleic acid interactions occurred under hydrophobic interactions, but not all ligands could bind to the model DNA molecules under these conditions. It is noticeable that ligands with amino substituents 2 and 4 in the R1 position show a stronger binding capacity in hydrophilic conditions (Figure 4A), as expected, since both mimic lysine, an amino acid with high DNA affinity. However, as the structures of both lysine mimetics are different, their binding abilities also differ when combined with the other ligands. Amino substituent 4 does not show strong binding behavior in hydrophilic conditions when conjugated with substituents 8 and 9. Conjugates of substituent 4 with amino substituent 8 comprise an overall structure composed of three aromatic rings from the triazine scaffold and the two ligand structures. This structure is more favorable for hydrophobic and stacking interactions than for electrostatic interactions, which are predominant in hydrophilic environments. Ligand 5/8 exhibited strong binding behavior. Substituent 8 (mimicking Gln/Asn) may contribute significantly to strong binding due to its amide group positioned at the edge of an aromatic ring, enabling hydrogen bond formation.

Binding under a hydrophobic environment is illustrated in Figure 4B. In these conditions, more ligands bound to DNA, and 25 out of 158 exhibited strong binding behavior. Ligands with substituent 1 (mimic of L-Ala/Gly) in the R1 position showed an improvement in binding to homo-oligonucleotide T in a hydrophobic mode; also, ligand with substituent 9 (mimic of Thr) in the R2 position showed higher binding profile. Ring stacking interactions and hydrophobic interactions are favored, so amines 3, 4, 5, and 8 may increase the binding of hydrophilic substituents 1 and 9. Amine 2 (a mimic of lysine) also exhibited strong binding behavior in a hydrophobic environment. This suggests that the high affinity of this Lys-mimic to nucleic acid bases is not solely driven by electrostatic interaction but may also involve van der Waals forces and potentially additional stacking interactions mediated by the triazine ring.

**Figure 5:**
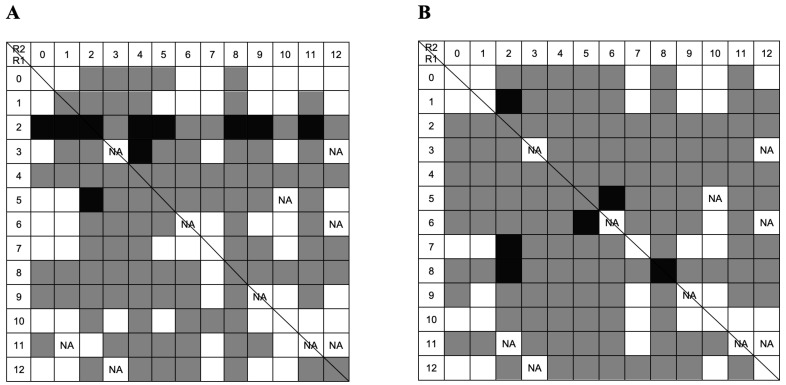
Summary of the FITC-based screening with 20 nt homo-oligonucleotide Poly G in: (**A**) hydrophilic conditions using 20 mM Tris-HCl pH 8.0 as the binding buffer; (**B**) hydrophobic conditions using 0.4 M ammonium sulfate in 20 mM Tris-HCl pH 8.0 as the binding buffer. Black squares indicate strong binders, gray squares indicate binders, and white squares indicate non-binders. NA: ligand not available. The line separates ligands that are obtained by adding the same substituents in reverse order on solid-phase synthesis [21].

FITC-labeled homo-oligonucleotide G (FITC-Poly G) was also screened to bind the same 158 ligands. When the incubation with FITC-Poly G was performed in a hydrophilic environment (Figure 5A), a lower number of non-binding ligands (49 vs. 67) was observed in comparison with FITC-Poly T. This result can be explained by the fact that the binding between guanine and amino acids, including lysine, is highly favored [23]. The same occurred in a hydrophobic binding environment. However, the results also show a lower number of strong binders when compared to binding to FITC-Poly T. A possible explanation is that FITC-Poly G oligonucleotide forms secondary structures such as guanine quadruplexes. Such quadruplexes originate when regions rich in guanine self-associate to form four-stranded structures [28]. This gives rise to a variety of structures involving one, two, or more DNA strands that can be oriented in many ways. The presence of such complexes may induce heterogeneity and affect the binding to solid-phase ligands.

Ligands with substituent 2 in the R1 position showed strong binding to FITC-Poly G under hydrophilic binding conditions, further demonstrating the high affinity between amine 2 and nucleic acid molecules. In a hydrophobic environment (Figure 5B), only a few ligands demonstrated strong binding to guanine, with ligands 1/2, 5/6, 6/5, 7/2, 8/2, and 8/8 being the exceptions. Amino substituents 5 and 8 have hydrophobic properties and the potential to form stacking interactions. Ligands with amine 2 also exhibited strong binding properties in a hydrophobic environment. Notably, ligands 5/6 and 6/5 displayed symmetry, and both displayed strong binding behavior under these conditions.

The results from the high-throughput FITC-based screening allowed the selection of ligands for testing in a microscale affinity chromatographic assay. Ligands were chosen based on their binding ability, symmetry, and unique combination of amino acid mimetics. The ligands selected based on screening results in hydrophilic conditions were as follows: 1/3, 3/5, 3/9, 4/3, 5/8, and 11/8. The ligands identified as strong binders in hydrophobic conditions included the following: 1/2, 1/8, 8/1, 2/2, 2/3, 5/11, 6/8, 8/6, 7/2, 8/2, 8/8, 8/11, 9/8, and 10/1. Lastly, two ligands, 5/6 and 6/5, were selected for their strong binding profiles in both environments. Ligands with amine 2 in either R1 or R2 were excluded from the group to be tested in hydrophilic conditions, as it has been previously shown that substituent 2 exhibits strong binding in these conditions (preliminary binding assessment). Oppositely, ligands containing substituent 2 that displayed strong binding in hydrophobic conditions were considered to be of interest to assess the role of this substituent in binding to various DNA molecules under conditions that do not favor electrostatic interactions.

### 2.3. Screening by Microscale Affinity Chromatography with Single-Stranded Oligonucleotides

#### 2.3.1. Screening with Thymine Homo-Oligonucleotide

The high-throughput FITC-based screening led to the selection of 22 ligands (out of 158) for screening by affinity chromatography in 0.2 mL (0.2 g) microscale columns containing ligand-derivatized resins with Poly-T homo oligonucleotide. The ligands were tested under conditions where each presented strong binding behavior. Not all results obtained in the chromatography assays were consistent with those obtained in the FITC-based screening. A total of 4 ligands out of the 22 tested emerged as false positives to Poly-T. Ligands 1/3, 3/9, 5/6 (in hydrophilic environment), and 10/1 (in hydrophobic conditions) presented intense fluorescence with the FITC-labeled Poly T homo-oligonucleotide but weak binding in the column chromatographic assay. False positives were previously described to occur when the FITC screening method is used [29] and can be attributed to experimental errors. Ligands 1/3, 3/9, and 5/6 have all hydrophobic substituents and, therefore, may not bind well to DNA molecules in hydrophilic conditions. Ligand 10/1 contains an aspartic acid mimic at the R1 position (which is negatively charged) and a small hydrophobic substituent (Ala/Gly) at the R2 position. Therefore, it is likely that binding under hydrophobic conditions will be weak.

Ligands 1/2, 7/2, 8/2, and 11/8 exhibited enhanced binding capabilities when evaluated through column chromatography, behaving as strong binders instead of binders (Table S1). This outcome aligns with previous expectations, given that ligands 1/2, 7/2, and 8/2 incorporate substituent 2 within their molecular structures. It has been demonstrated that this substituent significantly enhances binding affinity in both hydrophilic and hydrophobic environments. Additionally, amine 8, which mimics asparagine and glutamine, presents as a substituent capable of binding to DNA in both environments. This capability is attributed to its aromatic ring and amide group, which facilitate effective interactions with the target molecules.

#### 2.3.2. Screening with Guanine Homo-Oligonucleotide

The same set of 22 ligands was evaluated using a Poly G homo-oligonucleotide (Table S2). While ligands 5/8, 5/11, 6/8, and 8/6 demonstrated complete (100%) binding to thymine (Table S1), their binding affinity for guanine was significantly lower, with yields of approximately 42.8%, 31.2%, 33.8%, and 44.7%, respectively. This reduced binding efficiency may be explained by the presence of guanine secondary structures in the sample. To mitigate this issue, affinity chromatography could be performed at elevated temperatures to minimize the formation of such higher-order structures. Despite these challenges, ligands 1/3, 3/9, and 5/6 exhibited notably higher binding to the Poly G oligonucleotide. As previously discussed, amines 3 and 5 contain aromatic rings, which likely promote stronger π–π stacking interactions with the double-ring structure of guanine, as compared to the single-ring structure of thymine.

### 2.4. Screening by Microscale Affinity Chromatography with Double-Stranded Oligonucleotides (AT and GC)

Double-stranded DNA has a characteristic melting temperature at which the strands dissociate into single strands. Leveraging this property, pre-tested single-stranded DNA oligonucleotides were hybridized with their complementary strands. To verify duplex formation, melting curves were analyzed using a SYBR Green I-based real-time PCR assay [30]. The analysis of the melting curves showed that, as expected, double-stranded secondary structures were present in the solution after the hybridization procedure. When comparing the curves corresponding to double-stranded AT and GC with those of single-stranded Poly T and Poly G, a noticeable difference in fluorescence intensity was observed, particularly at lower temperatures. A defined peak was detected at around 63 °C, corresponding to AT impairment. For GC impairment involving all 20 bases, the peak appears at 78 °C.

**Figure 6:**
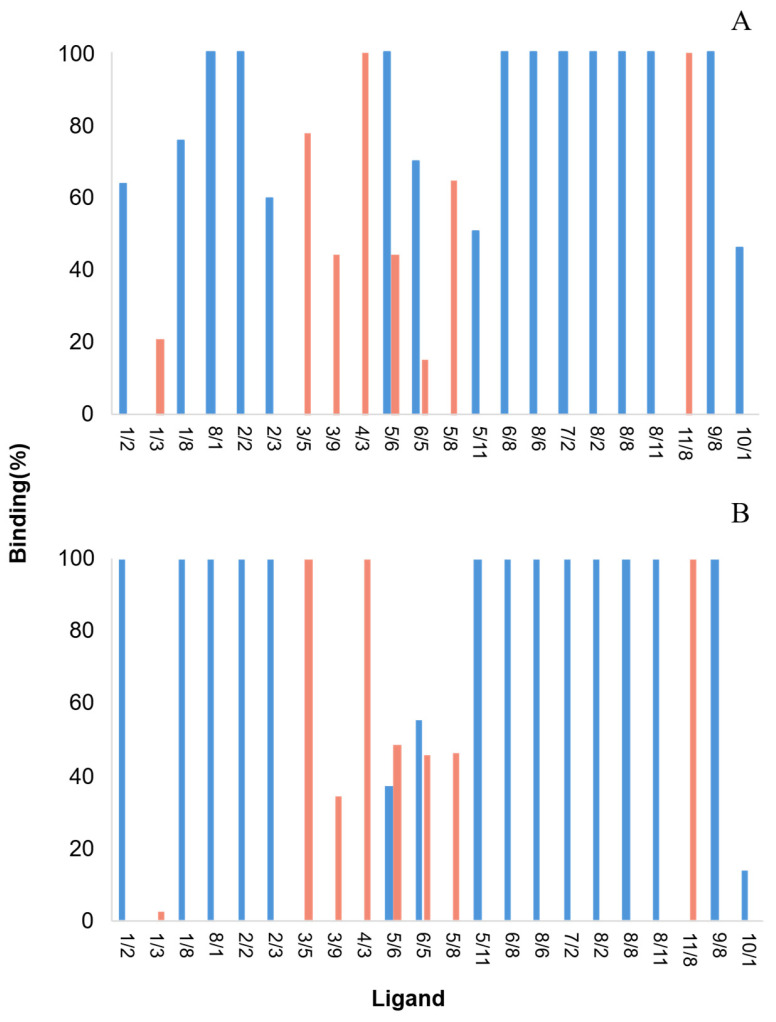
Comparison between the binding affinity of selected ligands towards (**A**) double-stranded AT, and (**B**) double-stranded GC oligonucleotides. Ligands were tested by microscale affinity chromatography in the conditions in which each selected ligand presented strong binding behavior towards homo-oligonucleotides in the FITC-based screening. The oligonucleotide mass loaded was 4.5 µg for AT. As for GC, the loaded mass was 2.55 µg from ligand 4/3 to ligand 6/5, and 3.95 µg from ligand 5/8 to 10/1. Blue bars: hydrophobic conditions using 0.4 M ammonium sulfate in 20 mM Tris-HCl pH 8.0 as the binding buffer. Orange bars: hydrophilic conditions using 20 mM Tris-HCl pH 8.0 as the binding buffer.

The 22 ligands that were selected in the previous FITC-based screening process were tested for their ability to bind to double-stranded oligonucleotide molecules (AT and GC). Of the 22 ligands, only 6 did not bind 100% of the double-stranded GC DNA. As for the AT pair, 11 ligands out of 22 did not bind 100% of the double-strand nucleotides (with yields ranging from 20.7% to 77.8%) (Figure 6). These results confirmed that some biomimetic affinity ligands exhibited specificity towards certain base pairs. It has been reported that the binding profile of some ligands may be affected by the obstruction of specific hydrogen-bond donors and acceptor atoms by the complementary nucleic acid. Therefore, ligands with an affinity for the DNA backbone may perform better [28]. The following screening step involved assessing the selected ligands’ ability to bind to a 6.0 kb plasmid by an affinity chromatographic assay.

### 2.5. Screening Assays Using Plasmid DNA

In preliminary assays using real DNA samples, the model plasmid (pVAX1/LacZ) showed very low binding to different ligands in microscale affinity chromatographic assays. It was therefore hypothesized that the resin volume was insufficient to retain large plasmid DNA molecules. Ligand 8/2, which was previously shown to be a strong binder of single- and double-stranded oligonucleotides under hydrophobic conditions, was used to test the binding of double-stranded and denatured plasmid. Samples of purified plasmid (without any RNA) were heated to 95 °C and denatured into their single-stranded form. In this case, a microscale affinity batch assay (0.2 g resin) was performed with the heated samples, maintaining the elevated temperature during binding. Three chromatography assays were performed: two positive controls using single-stranded Poly G and a hybridized GC pair, and a negative control composed of pure double-stranded plasmid DNA.

As shown in Table 2, ligand 8/2 was able to bind to single- and double-stranded oligonucleotides. Binding to plasmid DNA was not detected, in either its native or denatured form. Following these results, chromatographic assays for evaluating the potential of selected ligands to bind and purify pDNA were carried out in columns containing 2 g (instead of 0.2 g) of ligand-derivatized resins.

### 2.6. Purification of Plasmid DNA from a Clarified E. coli Crude Extract

Ligands 1/2, a mimic of a dipeptide Ala-Lys or Gly-Lys, and 2/3, a mimic of a dipeptide Lys-Tyr, were selected as initial candidates for a preliminary assessment of binding to plasmid DNA from an *Escherichia coli* crude lysate. Plasmid pVax1-LacZ, containing a GC content of 55% and an AT content of 45%, was used as a model pDNA. The impure extract contains plasmid DNA, RNA, and traces of genomic DNA. All the contaminants can potentially interact with the ligands. The most hydrophobic contaminant is single-stranded RNA, while the native double-stranded plasmid has the hydrophobic bases shielded inside the double helix. The crude *E. coli* extract underwent precipitation with ammonium sulfate, which removed some RNA.

The selection of these two ligands was guided by their specificity for different nucleotides, with the primary objective of enabling plasmid purification based on specific DNA sequences. Ligand 1/2 demonstrated the ability to bind to all GC double-stranded DNA but only 64% of AT, while ligand 2/3 exhibited similar behavior, binding 60% of AT, under hydrophobic conditions.

**Figure 7:**
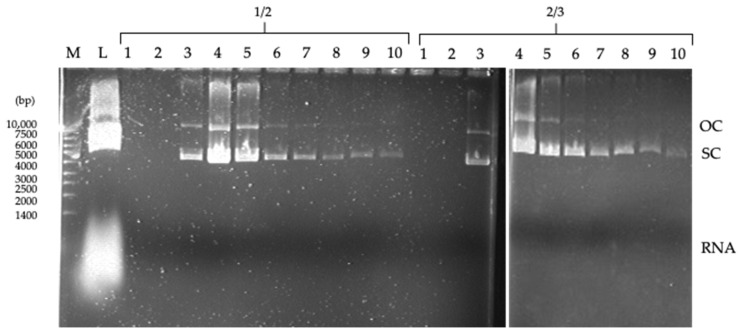
Agarose gel analysis of the samples (1–10) collected in the wash-through fraction with ligands 1/2 and 2/3 (in columns containing 2 mL of ligand-derivatized resin) using 0.4 M ammonium sulfate in 20 mM Tris-HCl pH 8.0 as the binding buffer. M–NZYDNA ladder II; L–Loaded sample (100 µL of clarified *E. coli* crude extract). The dragging marks in some lanes are due to the presence of ammonium sulfate.

The clarified extract was loaded onto columns containing 2 mL of moist gel (~2 g of ligand-derivatized resin). Samples of 200 µL were collected and analyzed by agarose gel electrophoresis. Figure 7 shows the results obtained with ligands 1/2 and 2/3 tested under hydrophobic conditions. A clear binding of the RNA molecules was observed with both ligands. Plasmid DNA is eluted during the wash-through step. Both isoforms are eluted simultaneously, with a clear tendency for the open circular isoform to elute in the first four fractions. As both ligands exhibit the same behavior, it is evident that substituent 2 greatly influences the binding, especially to the more hydrophobic RNA molecule. As previously noted, amine 2 has demonstrated the ability to engage in hydrophobic interactions with single-stranded nucleic acid molecules.

The results obtained with ligands 1/2 and 2/3 are similar to those obtained in the purification of plasmid DNA from *E. coli* clarified extracts by hydrophobic interaction chromatography (HIC) [31]. Purification of crude *E. coli* lysates by HIC results in a strong retention of RNA molecules by the resin, whereas the less hydrophobic plasmid DNA is excluded in the first fractions. Therefore, in a preliminary assessment, ligands 1/2 and 2/3 were identified as suitable candidates for purifying plasmid DNA free of RNA contamination, since this molecule remains strongly attached to the ligand-derivatized resins.

Chromatographic assays under hydrophobic conditions in a 6 mL agarose column with ligand 1/2, integrated into an ÄKTA Purifier system, confirmed its potential as an affinity ligand for plasmid DNA purification. However, an effective elution method for resolving both DNA isoforms has yet to be investigated. Competitive elution using amino acids, such as arginine or histidine, or other compounds with a high affinity for DNA molecules, could offer a promising alternative solution.

Future work will address more quantitative approaches to using selected ligands under optimized conditions for downstream process applications in plasmid DNA recovery.

## 3. Materials and Methods

### 3.1. Materials

All chemicals were of the highest purity available unless otherwise stated. Oligonucleotides were purchased from StabVida (Lisbon, Portugal) and are listed in Table 3 and Table 4. Sepharose CL-6B and PD-10 columns were purchased from Amersham-Pharmacia Biosciences (Milton Keynes, UK).

Plasmid pVAX1-LacZ was extracted from *E. coli* DH5α cells purchased from Invitrogen (Carlsbad, CA, USA). A cell bank was produced, and the cells were stored in sterile 20% (*v*/i) glycerol at −80 °C, in 1.5 mL tubes.

### 3.2. Fermentation

A pre-inoculum of *E. coli* DH5α harboring the 6.05 kb plasmid pVAX1-LacZ (Invitrogen, Carlsbad, CA, USA) was grown overnight in previously autoclaved (121 °C for 20 min) 100 mL shake flasks containing 30 mL of LB medium (Luria–Bertani–NaCl: 5 g/L; Tryptone:10 g/L; Yeast extract:5 g/L) from Sigma Aldrich (Darmstadt, Germany) with 30 μg/mL kanamycin, at 37 °C and 250 rpm, in an orbital shaker AGITORB 200 (Aralab, Lisbon, Portugal), up to an OD600 nm of 3. The appropriate amount of this culture was then used to inoculate 250 mL of LB medium (in 2 L Erlenmeyer flasks) at an OD_600 nm_ of 0.2. After adding 250 µL of 30 µg/mL kanamycin, the culture was incubated at 37 °C and 250 rpm for 6–7 h up to the late exponential growth phase (OD_600 nm_ ≈ 3). Cells were harvested by centrifugation in 500 mL flasks at 6000× *g* for 15 min at 4 °C in a Sorvall RC6 centrifuge with an SLA 3000 rotor (Thermo Fisher Scientific, Osterode, Germany). The supernatant was discarded, and the pellet was subjected to a standard alkaline lysis procedure [31].

### 3.3. Cell Lysis

The cell pellet was suspended in 8 mL of 25 mM Tris-HCl pH 8.0, with 50 mM glucose and 10 mM EDTA. After total suspension, the solution was passed to a 45 mL centrifuge tube, and 8 mL of 0.2N NaOH 1% (*w*/*v*) SDS) was gently added to start the alkaline lysis. The tubes were left to rest at room temperature for 10 min. The lysis was stopped using 8 mL of 5 M potassium acetate, 6.8 M glacial acetic acid, with gentle homogenization, and left resting on ice for 10 min [31]. The resulting lysate was centrifuged in an SS–34 rotor in a Sorvall RC6 centrifuge (Thermo Fisher Scientific, Osterode, Germany) at 20,000× *g* for 30 min at 4 °C to remove proteins, precipitated gDNA, and cell debris. To ensure the complete removal of cell debris, the resulting supernatant was transferred to a new tube and re-centrifuged at 20,000× *g* for 30 min at 4 °C. The lysate was then stored at −20 °C until further processing.

### 3.4. Concentration and Clarification of the Lysate

The lysate solution was precipitated with 0.7% (*v*/*v*) of isopropanol and left on ice for at least 2 h at 4 °C to precipitate all nucleic acids (pDNA, RNA, and traces of gDNA). The mixture was then centrifuged in a SS-34 rotor in a Sorvall RC6 centrifuge using the same settings as in the cell lysis step, and the supernatant was discarded. The pellet was washed with 2 mL of ethanol 70% (*v*/*v*) and centrifuged. The supernatant was discarded, and the tubes with the resulting pellet were inverted on top of absorbent paper to remove traces of ethanol. The pellet was suspended in 1 mL of Tris-HCl 20 mM pH 8.0, and the mixture was transferred to an Eppendorf tube. Remaining impurities, such as proteins and high molecular weight RNA, were precipitated by adding 0.337 g of ammonium sulfate (33.7% *w*/*v*) [31]. The mixture was homogenized and left on ice for 15 min, centrifuged at 20,000× *g* and 4 °C for 30 min (Eppendorf AG, Hamburg, Germany), and the resulting supernatant was stored at −20 °C.

### 3.5. Synthetic Affinity Ligand Library

A solid-phase ligand library composed of synthetic affinity ligands, each containing two commercially available amines that mimic amino acid side chains on a triazine scaffold, was synthesized as described in [21]. Epoxy-activated Sepharose CL-6B was suspended in distilled water, and ammonia 35% (*v*/*v*) was added. The gel was incubated for 12 h at 30 °C on a rotary shaker (200 rpm) and then thoroughly washed with distilled water on a sinter funnel. Aminated agarose (25 mmol amine/g moist weight gel, quantified by a TNBS method described in [32]) was suspended in a 50% (*v*/*v*) acetone–distilled water solution. This solution was maintained at 0 °C in an ice bath, and cyanuric chloride (in a proportion of five molar equivalents relative to the gel amination) dissolved in acetone was added portion-wise for 2 h with shaking and pH maintenance (neutral pH by addition of 1 M NaOH). The dichlorotriazinyl gel was divided into equal portions, and each portion reacted with a 2-fold molar excess of an aminated compound in an appropriate buffer (R1 substitution). Each R1-substituted gel was then reacted with a 5-fold molar excess of an aminated compound in an appropriate buffer (R2 substitution) [21].

### 3.6. Screening of Ligands Using DNA Single-Stranded Oligonucleotides Conjugated to FITC

A FITC-based method, adapted from [29], requiring very low amounts of reactants, conjugated FITC-oligonucleotide, and immobilized ligands, was used to test solid-phase immobilized ligands and identify strongly DNA-binding ligands, in different reaction conditions and with different single-stranded homo-oligonucleotides (Poly T and Poly G). Solid-phase affinity resins, containing 158 ligands from a combinatorial library, were distributed by four 96-well microplates (15 µL per well). The plates were centrifuged (Eppendorf 5810R, Eppendorf AG, Hamburg, Germany) for 2 min at 1430× *g*, and the supernatant (ethanol 20% *v*/*v*) was discarded. 100 µL of distilled water was added to each well, followed by centrifugation using the same conditions. 100 µL of regeneration buffer (0.1 M NaOH in 30% (*v*/*v*) isopropanol in distilled water) was added and centrifuged. The regeneration process was repeated. Distilled water (100 µL) was added, and the plate was subjected to centrifugation as before; the process was repeated twice. 100 µL of 20 mM Tris-HCl pH 8.0 (hydrophilic conditions) or 0.4 M ammonium sulfate in 20 mM Tris-HCl pH 8.0 (hydrophobic conditions) were added to each well. The plates were centrifuged for 2 min at 1430× *g*, the supernatant discarded, and the process repeated to ensure an effective equilibration step. After, 15 µL of 0.1 µM FITC-labeled oligonucleotides were added to each resin, and the mixture was incubated in the dark for 15 min, in the absence of light, with orbital agitation (TITRAMAX 1000, Heidolph Instruments, St. Louis, MO, USA). From this point on, all the procedures were performed in the dark. The wells were washed with 3 × 300 µL of the respective equilibration buffer (20 mM Tris-HCl pH 8.0 or 0.4 M ammonium sulfate in 20 mM Tris-HCl pH 8.0), centrifuged, and the supernatant was discarded. Finally, 200 µL of equilibration buffer was added to each well. The plates were placed under a fluorescence microscope (LEICA DMLB microscope, Leica Microsystems, Hessen, Germany), incorporating a digital camera Olympus DP10, an epi 100 lamp, and a WB filter (FITC excitation: 490 nm; FITC emission: 525 nm) to obtain high-resolution images. The pictures were analyzed using ImageJ software (version 1.50), and the fluorescence emission was qualitatively evaluated for each ligand.

### 3.7. Chromatographic Binding Assays

Each affinity resin (0.2 g ~ 0.2 mL of moist gel for screening of oligonucleotides (microscale assay) or 2.0 g ~ 2.0 mL of moist gel for the plasmid purification assays) was packed in a 4 mL (0.8 × 6 cm) PD-10 column. Each column was washed with 2 × 1 mL of regeneration buffer (NaOH (0.1 M) in 30% (*v*/*v*) isopropanol in distilled water), followed by distilled water. 10 mL of equilibration buffer (20 mM Tris-HCl pH 8.0 in hydrophilic experiments or 0.4 M ammonium sulfate in 20 mM Tris-HCl pH 8.0 in hydrophobic experiments) was added to the column, and 100 µL of sample containing selected oligonucleotides or *E. coli* lysate extract was loaded. Equilibration buffer was passed through the column (2 × 1 mL), and 200 µL samples were collected. The DNA concentration in the samples (load and collected fractions) was assessed using a nanodrop device (NanoValue Plus, GE Healthcare, Chicago, IL, USA) by measuring the absorbance at 260 nm. The percentage of DNA bound to the resin was calculated using the following equation:

(1) $$\text{Bound DNA}\left(\%\right)=\frac{\text{Mass of DNA loaded}-\text{Mass of DNA in collected fractions}}{\text{Mass of DNA loaded}}\times 100$$

The column was washed with 4 mL of regeneration buffer and 4 mL of distilled water, and stored at 4 °C, in ethanol solution (20% *v*/*v*), until further use.

### 3.8. Hybridization Assay with Homo-Oligonucleotides

To study the binding and specificity of selected ligands towards double-stranded DNA oligonucleotides, hybridization of single-stranded homo-oligonucleotides was performed. Each homo-oligonucleotide was paired with its natural base pair. Poly G (20 nt) was paired with Poly C (20 nt), and Poly T (20 nt) was paired with Poly A (20 nt). Two solutions were prepared in Eppendorf tubes, one containing 20 µL of 100 µM Poly A and 20 µL of 100 µM Poly T, and the other containing 20 µL of 100 µM Poly C and 20 µL of 100 µM Poly G. To both Eppendorf tubes, 10 µL of annealing buffer (10 mM Tris, pH 7.5–8.0, 50 mM NaCl, 1 mM EDTA) was added. The Eppendorfs were placed in a PCR thermocycler (Thermo Hybrid Biometra, Göttingen, Germany). The first step of the annealing protocol maintained the temperature at 95 °C for 5 min; in step two, the temperature gradually decreased from 95 °C to room temperature with a decrease of 0.03 °C per second. Hybridization between single-stranded oligonucleotides was assessed by melting curve analysis [30] using a SYBR^®^ Green I kit from Roche Diagnostics (Mannheim, Germany). Four qPCR capillaries were prepared, containing 15 µL of Milli Q water, 1 µL of MgCl_2_ solution, 2 µL of SYBR Green, and 2 µL of template solution, which contained one of the different homo-oligonucleotides or one of the annealed nucleotides (Poly G, Poly T, AT, GC); the hybridization program was run in a Roche PCR Lightcycler (Roche Diagnostics, Mannheim, Germany) with continuous acquisition of fluorescence emission data.

### 3.9. Agarose Gel Analysis

DNA analysis in agarose gels was performed using 1% agarose for plasmids or 4% agarose (SeaKem LE Agarose, Basel, Switzerland) for oligonucleotides in 1× TAE buffer. Separation was achieved at 90 V for 1 h (10 cm gels) or 120 V for 1.5 h (20 cm gels), followed by staining with 0.5 µg/mL ethidium bromide and imaging with a Stratagene EagleEye II system. NZYDNA Ladder II served as the size marker.

## 4. Conclusions

A triazine-based combinatorial library of synthetic affinity ligands was screened to identify candidates capable of binding nucleic acids for potential application in plasmid DNA purification. Initial results showed that a lysine mimic, 1,5-diaminopentane, effectively enhanced DNA-binding affinity when paired with various amino acid analogs. High-throughput screening using FITC-labeled thymine and guanine homo-oligonucleotides revealed distinct base preferences among the 158 ligands tested, with an unexpected stronger binding to thymine, likely due to a guanine-induced secondary structure. From this screen, 22 ligands were shortlisted and further evaluated using microscale affinity chromatography. Two lead candidates—dipeptide mimics of Ala-Lys/Gly-Lys and Lys-Tyr—demonstrated selective binding to double-stranded GC and AT oligonucleotides, and effectively purified plasmid DNA from *E. coli* lysates free of RNA contamination. These findings highlight the potential of triazine-scaffolded biomimetic ligands in nucleic acid (DNA or RNA) purification workflows. Future work will focus on using selected ligands under optimized conditions for downstream process applications in plasmid DNA recovery.

## Figures and Tables

**Table 1:** Structures of substituents of the triazine-based combinatorial ligand library and their corresponding analog amino acid side chains. In the solid-phase synthesis, subsequent substitution of each amine analog generates a ligand designated as x/y; x and y correspond to the numbered aminated compounds in the table, in the order in which R1 and R2 are substituted on the triazine ring. The negative control ligand, generally designated as 0/0, corresponds to the substitution of the two positions with an amine group (amine 0). Adapted from [22].

Number	Structure and Chemical Name	Analog of	Number	Structure and Chemical Name	Analog of
1	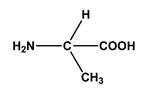 L-alanine	Alanine (Ala) Glycine (Gly)	7	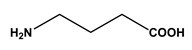 4-aminobutanoic acid	Glutamic acid (Glu)
2	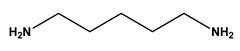 1,5-diaminopentane	Lysine (Lys)	8	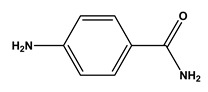 4-aminobenzamide	Glutamine (Gln) Asparagine (Asn)
3	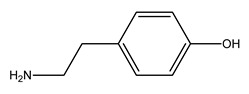 tyramine	Tyrosine (Tyr)	9	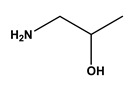 1-amino-2-propanol	Threonine (Thr)
4	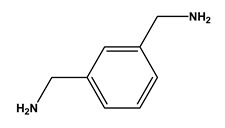 m-xylylenediamine	Lysine (Lys)	10	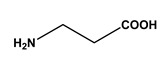 β-alanine	Aspartic acid (Asp)
5	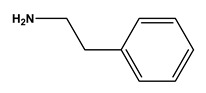 phenethylamine	Phenylalanine (Phe)	11	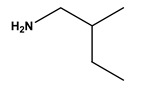 2-methylbutylamine	Isoleucine (Ile)
6	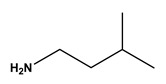 isoamylamine	Leucine (Leu)	12	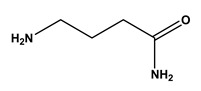 4-aminobutyramide	Glutamine (Gln)

**Table 2:** Results obtained in the binding of double-stranded and denatured plasmid DNA to ligand 8/2 in a microscale affinity assay (0.2 g resin). Single-stranded G and double-stranded GC oligonucleotides were used as positive controls.

	Mass Load (µg)	Bound Mass (µg)	Binding (%)
Single-strand homo-oligo (G)	2.5	2.5 ± 0	100
Double stranded oligo (GC)	4.0	4 ± 0	100
Double strand plasmid	1.6	0.03 ± 0.07	1.9
Single-stranded (denaturated) plasmid	1.6	0 ± 0.12	0

**Table 3:** FITC-labeled oligonucleotides used in the high-throughput FITC screening of the combinatorial library, and their respective sequences.

Name	DNA Sequence	Molecular Weight (g·mol^−1^)
FITC-Poly G	5′-FITC-GGGGGGGGGGGGGGGGGGGG-3′	7060
FITC-Poly T	5′-FITC-TTTTTTTTTTTTTTTTTTTT-3′	6560

**Table 4:** Oligonucleotides and their respective sequences used in chromatographic microscale affinity binding assays.

Name	DNA Sequence	Molecular Weight (g·mol^−1^)
MRS2rev	5′-TGCAAGCAACGCGTTAGCACATATGTG-3′	8309
MRS2fwd	5′-CATGCATTCGCGATTGGTCAAATTGGG-3′	8331
Poly A	5′-AAAAAAAAAAAAAAAAAAAA-3′	6202
Poly G	5′-GGGGGGGGGGGGGGGGGGGG-3′	6522
Poly T	5′-TTTTTTTTTTTTTTTTTTTT-3′	6022
Poly C	5′-CCCCCCCCCCCCCCCCCCCC-3′	5722

## Data Availability

Data are included within this article and its Supplementary Materials.

## Supplementary Materials

The following supporting information can be downloaded at: https://www.mdpi.com/article/10.3390/molecules30163423/s1, Table S1. Comparison of the results obtained in the FITC-based screening and the microscale affinity chromatographic assay, using single-stranded homo-oligonucleotide T (Poly T). Table S2. Comparison of the results obtained in the FITC-based screening and the microscale affinity chromatographic assay, using single-stranded homo-oligonucleotide G (Poly G).
