# Supplementary material for: Screening of a Combinatorial Library of Triazine-Scaffolded Dipeptide-Mimic Affinity Ligands to Bind Plasmid DNA
Source: Molecules. 2025 Aug 19;30(16):3423. doi: 10.3390/molecules30163423 (PMC12388571; doi:10.3390/molecules30163423)
Supplement: Supplementary file 1 [file molecules-30-03423-s001.zip › molecules-3760345-supplementary.pdf]

Screening of a Combinatorial Library of Triazine-Scaffolded Dipeptide-Mimic Affinity Ligands to Bind Plasmid DNA

João F. R. Belchior [1,2], Gabriel A. Monteiro [1,2], D. Miguel Prazeres [1,2] and M. Ângela Taipa [1,2,*]

[1] iBB—Institute for Bioengineering and Biosciences, and Associate Laboratory i4HB—Institute for Health and Bioeconomy at Instituto Superior Técnico, Universidade de Lisboa, Av. Rovisco Pais, 1049-001 Lisboa, Portugal
[2] Bioengineering Department, Instituto Superior Técnico, Universidade de Lisboa, Av. Rovisco Pais, 1049-001 Lisboa, Portugal
* Correspondence: angela.taipa@tecnico.ulisboa.pt

Supplementary Information

Table S1 - Comparison of the results obtained in the FITC-based screening and the microscale affinity chromatographic assay, using single-stranded homo-oligonucleotide T (Poly T). Each ligand was tested in optimal binding conditions (hydrophobic or hydrophilic), as determined in the FITC-based evaluation. The mass of homo-oligonucleotide applied in the assays using ligands 1/2 to 6/5 was 3.00 µg. From ligand 5/8 up to ligand 10/1, the mass of homo-oligonucleotide applied was 2.41µg.

Ligand	Conditions	FITC Result	Bind nucleotide mass(µg)	Binding (%)
**1/2**	Hydrophobic	B	3±0	100.0
**1/3**	Hydrophilic	SB	0.55±0.33	18.3
**1/8**	Hydrophobic	SB	3±0	100.0
**8/1**	Hydrophobic	SB	3±0	100.0
**2/2**	Hydrophobic	SB	3±0	100.0
**2/3**	Hydrophobic	SB	3±0	100.0
**3/5**	Hydrophilic	SB	3±0	100.0
**3/9**	Hydrophilic	SB	0.48±0.21	16.0
**4/3**	Hydrophilic	SB	3±0	100.0
**5/6**	Hydrophobic	SB	3±0	100.0
**6/5**	Hydrophobic	B	3±0	100.0

**5/6**	Hydrophilic	B	0.26±0.14	8.7
**6/5**	Hydrophilic	B	1.76±0.17	58.7
**5/8**	Hydrophilic	SB	2.41±0	100.0
**5/11**	Hydrophobic	SB	2.41±0	100.0
**6/8**	Hydrophobic	SB	2.41±0	100.0
**8/6**	Hydrophobic	SB	2.41±0	100.0
**7/2**	Hydrophobic	B	2.41±0	100.0
**8/2**	Hydrophobic	B	2.41±0	100.0
**8/8**	Hydrophobic	SB	2.41±0	100.0
**8/11**	Hydrophobic	SB	2.41±0	100.0
**11/8**	Hydrophilic	B	2.41±0	100.0
**9/8**	Hydrophobic	SB	2.41±0	100.0
**10/1**	Hydrophobic	SB	0±0.12	0.0

Table S2 - Comparison of the results obtained in the FITC-based screening and the microscale affinity chromatographic assay, using single-stranded homo-oligonucleotide G (Poly G). Each ligand was tested in optimal binding conditions (hydrophobic or hydrophilic), as determined in the FITC-based evaluation. The mass of homo-oligonucleotide applied in the assays using ligands 1/2 to 6/5 (hydrophobic) was 3.00 μg. From ligand 5/6 (hydrophilic) up to ligand 10/1, the mass of homo-oligonucleotide applied was 3.11 μg.

Ligand	Conditions	FITC Result	Binding nucleotide mass (μg)	Binding (%)
**1/2**	Hydrophobic	SB	3±0	100.0
**1/3**	Hydrophilic	B	0.98±0.09	32.7
**1/8**	Hydrophobic	B	3±0	100.0
**8/1**	Hydrophobic	B	3±0	100.0
**2/2**	Hydrophobic	B	3±0	100.0
**2/3**	Hydrophobic	B	3±0	100.0
**3/5**	Hydrophilic	B	3±0	100.0
**3/9**	Hydrophilic	B	1.76±0.43	58.7

**4/3**	Hydrophilic	B	3±0	100.0
**5/6**	Hydrophobic	SB	3±0	100.0
**6/5**	Hydrophobic	SB	3±0	100.0
**5/6**	Hydrophilic	B	1.33±0.21	42.8
**6/5**	Hydrophilic	B	0.99±0.06	31.8
**5/8**	Hydrophilic	B	1.33±0.14	42.8
**5/11**	Hydrophobic	B	0.97±0.11	31.2
**6/8**	Hydrophobic	B	1.05±0.06	33.8
**8/6**	Hydrophobic	B	1.39±0.16	44.7
**7/2**	Hydrophobic	SB	3.11±0	100.0
**8/2**	Hydrophobic	SB	3.11±0	100.0
**8/8**	Hydrophobic	SB	3.11±0	100.0
**8/11**	Hydrophobic	B	3.11±0	100.0
**11/8**	Hydrophilic	B	3.11±0	100.0
**9/8**	Hydrophobic	B	3.11±0	100.0
**10/1**	Hydrophobic	NB	0±0.03	0.0
